# A Deep Sequencing Approach to Uncover the miRNOME in the Human Heart

**DOI:** 10.1371/journal.pone.0057800

**Published:** 2013-02-27

**Authors:** Stefanos Leptidis, Hamid el Azzouzi, Sjoukje I. Lok, Roel de Weger, Serv Olieslagers, Natasja Kisters, Gustavo J. Silva, Stephane Heymans, Edwin Cuppen, Eugene Berezikov, Leon J. De Windt, Paula da Costa Martins

**Affiliations:** 1 Department of Cardiology, CARIM School for Cardiovascular Diseases, Faculty of Health, Medicine and Life Sciences, Maastricht University, Maastricht, The Netherlands; 2 Department of Pathology, University Medical Center Utrecht, Utrecht, The Netherlands; 3 Hubrecht Institute, Royal Netherlands Academy of Sciences, Utrecht, The Netherlands; Leibniz-Institute for Arteriosclerosis Research at the University Muenster, Germany

## Abstract

MicroRNAs (miRNAs) are a class of non-coding RNAs of ∼22 nucleotides in length, and constitute a novel class of gene regulators by imperfect base-pairing to the 3′UTR of protein encoding messenger RNAs. Growing evidence indicates that miRNAs are implicated in several pathological processes in myocardial disease. The past years, we have witnessed several profiling attempts using high-density oligonucleotide array-based approaches to identify the complete miRNA content (miRNOME) in the healthy and diseased mammalian heart. These efforts have demonstrated that the failing heart displays differential expression of several dozens of miRNAs. While the total number of experimentally validated human miRNAs is roughly two thousand, the number of expressed miRNAs in the human myocardium remains elusive. Our objective was to perform an unbiased assay to identify the miRNOME of the human heart, both under physiological and pathophysiological conditions. We used deep sequencing and bioinformatics to annotate and quantify microRNA expression in healthy and diseased human heart (heart failure secondary to hypertrophic or dilated cardiomyopathy). Our results indicate that the human heart expresses >800 miRNAs, the majority of which not being annotated nor described so far and some of which being unique to primate species. Furthermore, >250 miRNAs show differential and etiology-dependent expression in human dilated cardiomyopathy (DCM) or hypertrophic cardiomyopathy (HCM). The human cardiac miRNOME still possesses a large number of miRNAs that remain virtually unexplored. The current study provides a starting point for a more comprehensive understanding of the role of miRNAs in regulating human heart disease.

## Introduction

Beginning with the discovery of lin-4 in C. elegans in 1993 [1] and followed by let-7 in 2000 and its conservation amongst species [2], the importance and universal effect of microRNAs (miRNAs) have provoked an explosive field of research. miRNA genes, encoded by the genome, are a class of noncoding RNAs of 21–23 nucleotides (nt) in length, which derive from single-stranded RNA precursors [3]. They negatively regulate gene expression by direct binding to the 3′ untranslated region (3′ UTR) of a target mRNA, leading to its translational repression and/or deadenylation. Target recognition is also influenced by the secondary structure of regions surrounding the target sequence and the extent of seed sequence complementarity influences whether the miRNA/mRNA interaction will result in repression and/or deadenylation of the target mRNA. miRNA conservation among species, from plants to animals, as well as their vast biological importance has been firmly established in the past decade [4]. miRNAs are often grouped into families when sharing identical seed sequences and similar mature sequences, most likely also targeting common mRNAs, to different extents [5]. miRNAs display a characteristic spatial, temporal and tissue-specific expression profile and have great impact as key regulators in cell differentiation, growth [6], and apoptosis [7]–[9].

In the cardiovascular field, several studies have demonstrated the involvement of miRNAs in a number of physiological and pathological conditions [10]–[13]. Changes in cardiac miRNA expression levels have been associated to cardiac stress and development of cardiac hypertrophy not only in mice, as a result of transverse aortic constriction (TAC) [14], myocardial infarction and specific transgene expression [14]–[18], but also in human end-stage heart failure [15], [19]. Besides their specific expression patterns, miRNAs also exhibit remarkable biostability, allowing their detection in human plasma and serum, and suggesting that miRNAs may become relevant biomarkers in heart failure [13], [20].

Taking into account that miRNAs can possibly regulate at least 30% of human protein-coding genes, bioinformatics prediction algorithms in conjunction with biologic validation methodologies initially estimated the existence of roughly 35 miRNAs – a number that has kept rising ever since [21], [22]. Currently, more than twenty-one thousand miRNAs are annotated in miRBase v19.0 [5], with the total number of experimentally validated human miRNAs reaching approximately two thousand. While microRNA expression profiles resulting from different cardiac stresses display, to some extent, overlaps and suggesting common responses to different stimuli, there are also miRNAs that show a unique expression profile related to a specific cardiac stress. Since the seed sequences differ between different miRNA families, each family can regulate a different set of mRNAs. This concept has led to the perception that cardiac miRNA expression profiles may represent novel and specific signatures of disease, highlighting a network of target mRNAs with a central role in cardiovascular disease. In fact, a hallmark of cardiac hypertrophy and heart failure is a profound alteration of the myocardial transcriptome landscape, including mRNAs, miRNAs, long noncoding RNAs and other RNA species [23], [24], suggesting that changes in the transcriptome homeostasis are critically associated to the disease process.

For the reasons mentioned above, miRNA profiling has become of great interest in diverse research areas of biology and medicine, and is mostly defined as a measurement of the relative abundance of a set of miRNAs, ranging from a group of several miRNAs of specific biological relevance to comprehensive profiling of all miRNAs in a specific species. However, evaluating such small RNA molecules implies some intrinsic challenges. Whereas the application of DNA microarray technology to miRNA expression profiling offers advantages in terms of comparative capabilities, miRNA arrays do require a complicated procedure due to the size of miRNAs and the lack of a conserved 3-prime end for easy sample labeling, in conjunction with the fact that they frequently lead to process-related systematic biases. Despite the development of a number of different miRNA array platforms [25], [26], the requirement of prior sequence information used for probe design limits the depth of microarray expression profiling to discovery of annotated, experimentally validated and highly abundant transcripts. In contrast, large-scale parallel sequencing approaches along with custom computational pipelines have been carried out using different platforms [27], [28] to discover small non-coding RNA species abundance and existence in an unbiased manner in organisms such as plants, mice and primates [29]–[31].

Here, we report the use of large-scale sequencing as a technological RNA discovery platform to: 1) catalog the miRNAs expressed in the non failing and failing heart and 2) to quantify the miRNA content in the non-failing human heart and relative changes in the miRNOME in hearts from patients with dilated cardiomyopathy (DCM) from non-ischemic origin or hearts from patients with familial hypertrophic cardiomyopathy (HCM).

## Materials and Methods

### Human Subjects

In this study, sixteen (16) human subjects were included, from which four (4) were refused donor hearts (controls) and the other twelve (12) were hearts from patients with refractory end-stage heart failure. The heart failure patients were divided in two groups: seven (7) patients that were diagnosed with dilated cardiomyopathy (DCM), and five (5) patients diagnosed with hypertrophic cardiomyopathy (HCM). Characteristics of these patients are summarized in **Table S1**. All DCM patients were treated with a left ventricular assist device (LVAD, Heart-mate, Thoratec, Pleasanton, California) as a bridge to transplantation and were in NYHA class IV at the time of LVAD implantation. The myocardial biopsy at time of LVAD implantation consisted of the LV apical core removed during implantation (diameter was about 1 cm). In patients diagnosed with HCM, biopsies were taken immediately after explantation of the heart. From all biopsies half was immediately frozen in liquid nitrogen and the other half was directly fixed in buffered formalin and embedded in paraffin. Control tissue was taken from the left ventricle of non-used donor hearts. Informed consent to participate in this study was obtained from all patients and individual written permission using standard informed consent procedures and prior approval of the Medical Ethics Committee of the University Medical Center Utrecht, was obtained.

### Deep Sequencing

Myocardial samples from the three groups (control, DCM and HCM) were used to generate high-titer small RNA libraries by Vertis Biotechnology AG (Freising-Weihenstephan, Germany). Briefly, the small RNA fraction from human left ventricular free wall of patients with end-stage heart failure was isolated using the mirVana microRNA isolation kit (Ambion). Sample preparation for parallel sequencing was performed as described [32]. In short, sample enrichment was done by excision of the 15 to 30 nt fraction from a polyacrylamide gel, followed by cDNA synthesis with poly(A)polymerase for the addition of poly A-tails in the RNA molecules of this fraction. A synthetic RNA adapter was then ligated to the 5′ phosphate of the miRNAs. This was followed by first strand cDNA synthesis using an oligo(dT)-linker primer and M-MLV-RNase H-reverse transcriptase. cDNA was PCR-amplified with adapter- specific primers and used in single-molecule sequencing. Massive parallel sequencing was performed by 454 Life Sciences (Branford, USA) using the Genome Sequencer 20 system.

### Computational Analysis of Cloned Small RNAs Sequencing Reads

Analysis of primary data was performed using miR-Intess software (http://www.internagenomics.com), as previously described [33]. Following adapter sequence trimming, MEGABLAST software [34] was used to map inserts of 18 nt and longer, to the genome (ncbi36). Genomic loci annotations retrieved from the Ensembl database (homo_sapiens_core_53_36o) were used to classify aligned reads. Only reads aligned to intergenic or intronic regions, including simple and trf repeats, were used for further analysis by utilizing the discovery and annotation part of miR-Intess. Overlapping reads were grouped into blocks and were further analyzed by using the RNAshapes program [34], [35] to identify hairpin structures with the abstract shape “[]” and one arm containing a read loci, in sliding windows of 80,100 and 120 nt. Classification of the hairpins into three confidence levels (confident, candidate, non-confident) was performed after additional analysis of a number of parameters. These parameters included number, length and hairpin location of reads, start and end point variations, GC content, Drosha/Dicer signatures, randfold value and annotation of homologous hairpins in other genomes. Finally, annotations were used to split the positive hairpins into known or novel miRNAs.

### (microRNA) Real-time PCR

Primers were designed to detect mouse and human transcripts for 3615376, 5243049 and s2154. Sequences are depicted in **Table S2**. RNA (1 µg) from mouse or human tissue was isolated with TRIzol reagent (Invitrogen) and reverse-transcribed with the miScript Reverse Transcription Kit (Qiagen). Real-time PCR was performed on 1 ng of cDNA using miScript SYBR Green PCR Kit (Qiagen), on a BioRad iCycler (Biorad). To determine transcript quantity, the relative Ct method was used, where the amount of target normalized to the amount of endogenous control (U6) and relative to the control sample is given by 2–ΔΔCt [36]. Expression levels of all other miRNAs were determined by using a TaqMan MicroRNA Reverse Transcription Kit and MicroRNA assay (Applied Biosystems). The small nuclear RNA U6B was used as an internal control (RNU6B), and the sequence detection system ABI Prism 7900HT (Applied Biosystems) was used for read-out. The relative miR quantity was determined using the comparative Ct method.

### Stress Markers Real-time PCR

Primers were designed to detect human transcripts for natriuretic peptide A, (*nppa*), natriuretic peptide B (*nppb*) and glyceraldehyde 3-phosphate dehydrogenase (*gapdh*). Sequences are depicted in **Table S2**. RNA (1 µg) from human cardiac tissue was reverse-transcribed using Superscript II reverse transcriptase (Invitrogen) and 10 ng of cDNA were used for the analysis. Real-time PCR was performed on a BioRad iCycler (Biorad) using SYBR Green. Transcript quantities were compared using the relative Ct method, where the amount of target normalized to the amount of endogenous control GAPDH and relative to the control sample is given by 2–ΔΔCt.

### Statistical Analysis

The results are presented as mean ± standard error of the mean (s.e.m). Prism software (GraphPad Software) was used to perform all statistical analyses. For the correlation analysis, Reads data were log10-transformed and used to calculate the Pearson’s coefficient of correlation (r^2^). For comparison of two experimental groups ANOVA followed by a Student *t* test was used. Differences were considered significant when *p*<0.05.

## Results

To assess the number of expressed miRNAs in the human heart, both in physiological (non-failing) as well as in pathophysiological (hypertrophic and dilated cardiomyopathy) conditions, massive parallel sequencing technology was performed using an established small RNA cloning approach and 454 Life Sciences sequencing technology, as has been previously described [37]. As the minimum read length was 18 nucleotides (nt) increasing to a maximum of 30, the total number of raw reads obtained from small RNA libraries was ∼250 million, of which ∼28 million reads were mapped to the human genome (ncbi36 assembly).

**Figure 1:**
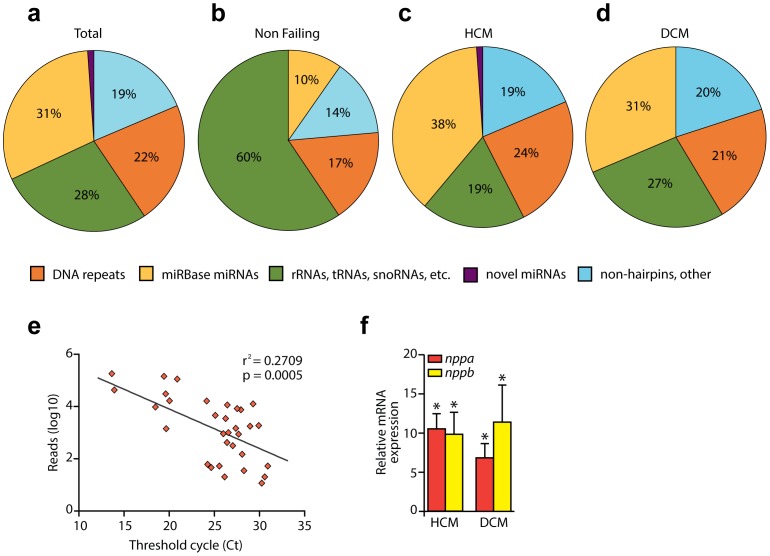
RNA content in the non-failing and failing human heart. A , Composition of global libraries, **B**, non-failing, **C**, hypertrophic cardiomyopathy (HCM), and **D**, dilated cardiomyopathy (DCM). **E**, Correlation of number of reads with cycle threshold (Ct) values obtained by real time quantitative PCR analysis of expression of 4 different microRNAs (miR-199a, miR-199b, miR-143 and miR-145). Data pairs are represented individually, independent of the miRNA analyzed. **F**. Real time quantitative PCR analysis of cardiac stress markers (nppa and nppb) in DCM and HCM samples.

Approximately 22% of the total mapped reads constituted of DNA repeat-related regions. A large fraction (28%) qualified to other non-coding RNA fraction including rRNAs, tRNAs, small nuclear RNAs (snRNAs), small nucleolar RNAs (snoRNAs) as well as small interfering RNAs (siRNAs). The sub-fraction of tRNAs was the most prominent one, accounting for 78% of the non-coding RNA fraction. The number of perfectly aligned miRBase annotated miRNAs was 563, accounting for 31% of the total reads (**Figure 1a**), yet a small percentage (0.02%) of the sequences indicated to potentially harbor novel miRNAs. The remaining reads consisted of other hairpins, non-hairpin RNAs or repeat-associated small interfering RNAs (rasiRNAs) (**Figure 1a**) [38]. The libraries exhibited considerable differences in these percentages, when comparing the physiological with the pathophysiological groups. Strikingly, there was a clear upregulation of the amount of miRBase-annotated miRNAs in the pathophysiological groups (mean of 35% compared with 10% of the physiological groups) whilst the differences within the disease groups were minor as shown in **Figure 1b–d**.

Since it has been previously shown that relative abundance of small RNAs can in general be reflected by their cloning frequency [39], the potential relationship between the number of reads and the normalized cycle threshold (Ct) values, obtained by quantitative PCR (qPCR), was evaluated in a correlation assay. Four different miRNAs were tested (miR-199a, miR-199b, miR-143, miR-145), for two NF, three DCM and three HCM samples. Each pair number of reads/Ct value obtained was seen as an independent data pair, regardless of the miRNA. As seen on **Figure 1e**, plotting the relation between the number of reads identified by deep sequencing and the normalized Ct values resulted in a significant correlation (n = 32, r^2^ = 0.2709, *p* = 0.0005), suggesting that read number could be used as an abundance indicator. In addition, increased expression of cardiac stress markers such as *nppa* and *nppb* in both the DCM and HCM samples confirm the integrity and relevance of the samples used in this study (**Figure 1f**).

**Figure 2:**
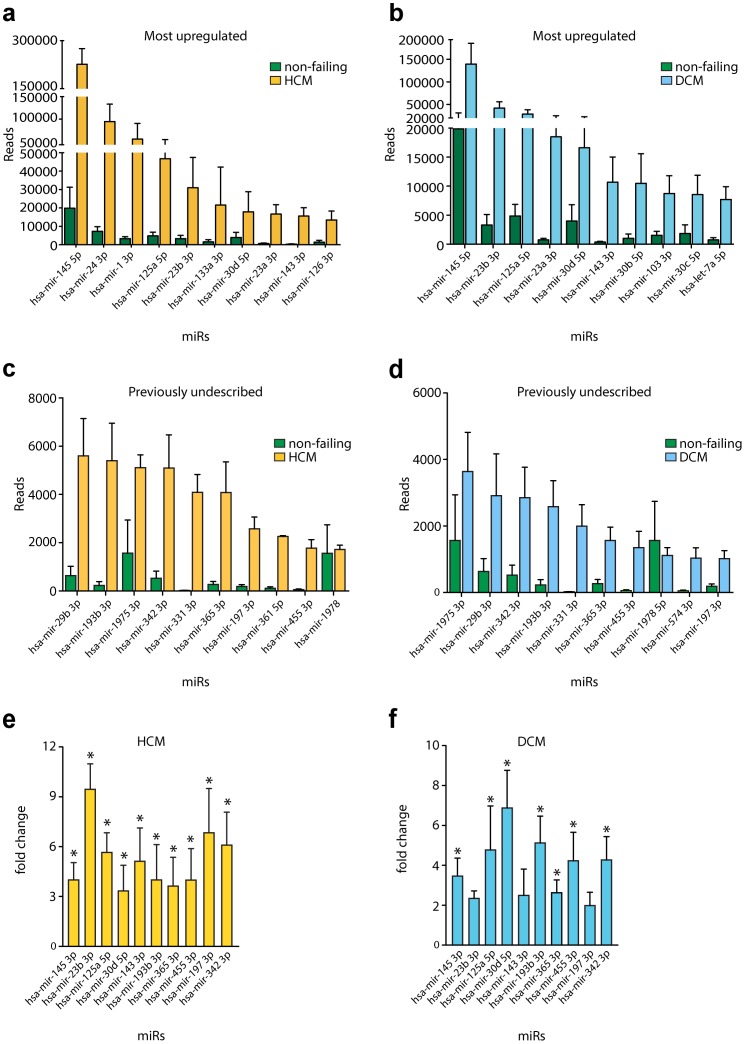
Hypertrophic cardiomyopathy (HCM) and dilated cardiomyopathy (DCM) present different microRNA expression patterns. Expression pattern of the 10 most upregulated microRNAs that have been previously related to cardiovascular disease and/or described in literature (**A**, **B**) or not (**C**, **D**), in the HCM and DCM libraries. **E**, **F**, Real time quantitative PCR analysis to validate results from **A**–**D**.

In order to further fine tune the expression profile of the miRBase annotated miRNAs, we employed a threshold in our data set, further analyzing all the miRNAs that showed a number of reads greater or equal to 10% lower than the number of reads of hsa-miR-195. miR-195 was chosen as threashold due to its relevant regulatory function during pathological cardiac remodeling despite its relatively low expression levels in the heart [15]. miR-92a has been previously shown to have a clear biologic function [40] in cardiovascular disease, and it was represented by a very low number of reads in our platform (mean of 20 reads for NF and ±200 in disease states), indicating the high sensitivity of this approach. Considering previously performed profiling studies of miRNAs in heart failure, the implemented cut-off returned a total number of 303 miRNAs with a subset of 162 of these miRNAs previously undescribed and unrelated to cardiovascular disease in any other profiling study (**Table S3**). The expression pattern of the 10 most abundantly expressed miRNAs in our study for HCM (**Figure 2a**) and DCM (**Figure 2b**) reveals a set of miRNAs that have been previously described to play an important role in CVD either by promoting (miR-23a [8], miR24 [41]) or inhibiting cardiac hypertrophic growth (miR-1 [16], [42] and miR-133a [14]).

**Figure 3:**
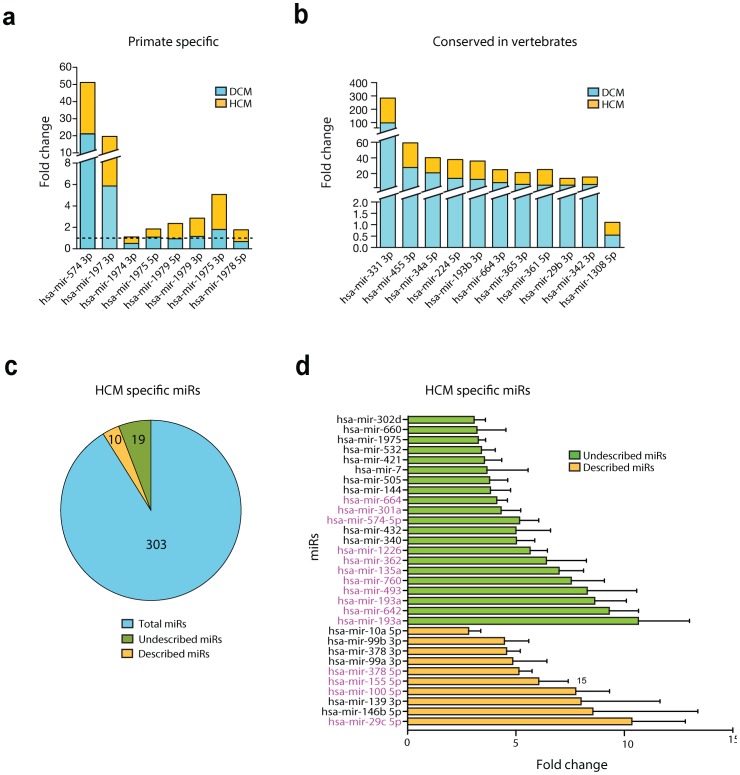
Hypertrophic cardiomyopathy-specific microRNA expression pattern. From the most differentially regulated microRNAs in the DCM and HCM libraries, 8 are specific for primates and conserved between primates (**A**) and 11 are highly conserved amongst vertebrates (**B**). C**,** From a total of 332 microRNAs detected in the HCM library, 29 were HCM-specifc, from which 10 had been previously described in profiling studies and 19 remain to be described. **D**, Relative abundance of HCM-specific microRNAs that were detected, or not, in previous profiling studies. MicroRNAs that are significantly expressed between HCM and DCM libraries are depicted in purple.

The 10 most differentially expressed miRNAs in HCM (**Figure 2c**) and DCM patients (**Figure 2d**) reveal a set of miRNAs that could have a potential biological function in cardiovascular disease but have been so far overlooked. Increase in read number correlated with increased miRNA expression levels for the miRNAs tested in HCM and DCM samples by quantitative PCR (**Figure 2e and 2f**). Interestingly, a number of these miRNAs were conserved among primates, as shown in **Figure 3a**, with limited potential for genetic studies in mouse models due to the lack of existing research tools. However, there was also a clear set of miRNAs that showed high conservation amongst vertebrates (**Figure 3b**). This subset can be readily analyzed using existing genetic technologies to verify the importance and function of these miRNAs in cardiovascular disease. Nonetheless, these data reveal the involvement and hidden potential of previously undescribed miRNAs in cardiovascular disease.

Identifying potential miRNAs that show specificity on either etiology would be significant for the discovery of novel diagnostic tools, early patient stratification as well as targeted therapy of both conditions. Investigating this potential, we applied a threshold of at least 3-fold differential expression, compared to the non-failing libraries for all miRNAs. Ten (10) out of the 141 miRBase miRNAs that have been previously described in other profiling studies, as well as 19 out of 162 miRBase miRNAs that were not, showed potential hypertrophic cardiomyopathy specificity, when compared with the non-failing and dilated cardiomyopathy samples (**Figure 3c,d**), paving the way for more etiology-specific studies.

**Figure 4:**
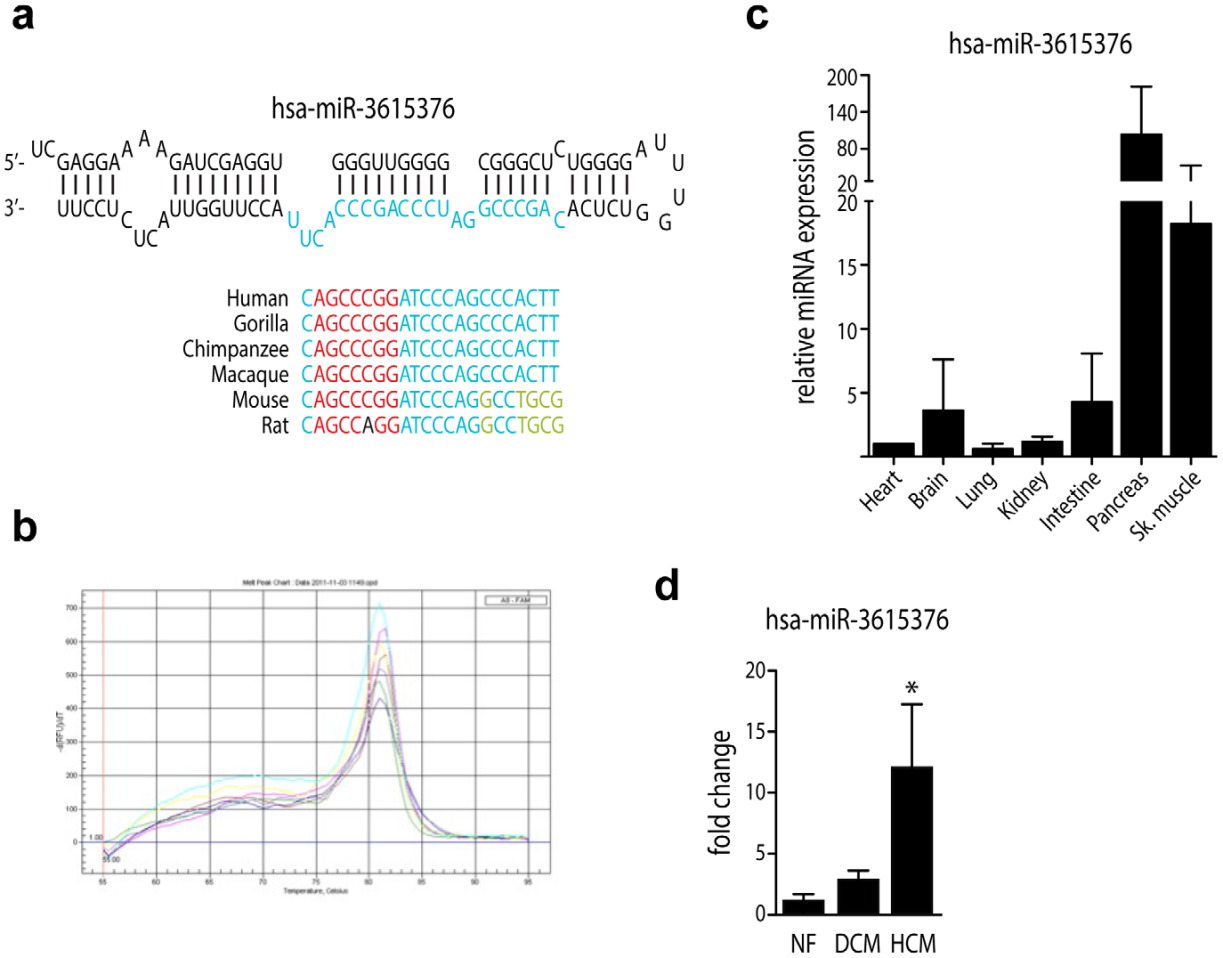
Identification of a novel microRNA. A, Schematic representation of the conservation of a novel microRNA, miR-3615376 among different species. **B, C,** Real-time quantitative PCR analysis of miR-3615376 expression in different mouse tissues. **D**, Expression pattern of miR-3615376 in the DCM and HCM libraries.

Analysis of the sequences also revealed a large number of non-annotated sequences that are encoded by intronic or intergenic regions with levels of conservation varying from within primates to vertebrates. Advanced analysis of these potentially novel miRNAs, through our computational pipeline, yielded a number of approximate 77 high-probability novel miRNAs as well as ∼3.500 low-probability novel miRNAs (probability defined by the total number of parameters met). Although these subsets exhibited extremely low cloning frequencies, in the range of 1–1500, the existence of at least 1 novel miRNA, has-miR-3615376, (**Figure 4a**) was experimentally verified by quantitative PCR on a variety of mouse tissues (**Figure 4b,c**). This miRNA, which has very recently been annotated in miRBase as hsa-miR-3940, is expressed in various mouse tissues and although at relative low levels, also expressed in cardiac tissue. Expression of miR-3615376 is elevated in cardiac disease, with a significant change in HCM (**Figure 4d**).

## Discussion

The main purpose of this study was the cloning, sequencing and identification of the global set of expressed mature miRNAs (the miRNOME) in the human heart, both in failing as well as in non-failing situations, rather then a quantitative comparison between those two situations. The resulting miRNA inventory can be used to further investigate potentially important regulators that have been so far overlooked by previous studies.

Although deep sequencing is emerging to be the method of choice for miRNA discovery and genome annotation using non-model organisms, profiling inconsistencies for known miRNA levels such as miR-1 and miR-133a, show that microarrays are often a preferable choice for miRNA profiling in model organisms with well-annotated genomes, due to their robust sample processing and analysis pipelines. In fact, while many miRNAs which regulation has been considered a signature of heart failure (miR-133a, miR-1, miR-21, miR-214, miR-212, miR-29, miR-129, miR199a) [18], [43]–[51], were confirmed by deep sequencing to be differentially regulated in HCM, only a few could be confirmed using this technique in DCM (miR-214, miR-129). Although we observed a much higher variability between samples in the DCM group then in HCM, which in combination with relative low group size resulted in no significant effects, deep sequencing in DCM samples did show increased read numbers for most of the heart failure hallmark miRNAs. Furthermore, our study identified many miRNAs that are reported here for the first time as differentially regulated in cardiac disease, strengthening the role of microRNAs in regulating the biological and molecular processes sustaining pathological remodeling of the heart. miR-145, one of the most abundant miRNAs in the heart according to our deep sequencing data, has not been previously related to pathological left ventricular remodeling. This miRNA is involved in smooth muscle cell fate and plasticity [52] where it functions to regulate the quiescent versus proliferative phenotype of smooth muscle cells. Regarding its role in the heart, miR-145 seems to be involved in cardiac remodeling following acute myocardial infarction, where sustained downregulation of myocardial pri-miR-145 expression was observed. [53] More recently miR-145 has been implicated in the development of pulmonary arterial hypertension (PAH), [54] since it is dysregulated in mouse models of PAH and its dowregulation is protective against the development of the disease. Here we report a significant increase of miR-145 expression both in DCM and HCM, confirmed both by deep sequencing and qPCR analysis and suggesting a role for this miRNA in left ventricle pathological remodeling. However, further investigation is needed to shed new light on the exact role of this miRNA, and many others, and the molecular pathways it regulates in the heart.

Nevertheless, due to the discovery power of high-throughput sequencing, even after applying our robust cutoff, we were able to identify a very considerable set of miRNAs (162) that could potentially be important in cardiovascular disease. Moreover, miRNAs that overlap with other non-coding RNAs such as the large class of tRNAs and the snoRNAs, were readily identifiable and excluded from further analysis, preventing erroneous annotation. Despite the debatable physiological relevance of low abundant miRNAs and their large representation in our study, their existence could prove to become significant in the development of novel-miRNA regulatory pathways. Our analysis also yielded a number of miRNAs that exhibit an etiological (hypertrophic versus dilated cardiomyopathy) specific expression background, which could be used as basis for further research to unveil the various pathways leading to different cardiovascular disease outcome. Finally, a number of novel miRNAs were detected. These novel sequences were missed by conventional analysis probably because they tend to be expressed at low levels in the heart and are, most likely, located within unannotated regions in the genome. Examples of studies where novel miRNAs were identified by parallel sequencing are in embryonic stem cells, leukemia and several types of cancer [34], [55]–[57]. The low expression levels of novel miRNAs could be effectively detected due to the high sensitivity of deep sequencing, which also suggests that the most abundant miRNAs have already been identified by conventional techniques such as array-based. Despite the low levels of expression for miR-3615376 (currently annotated as miR-3940), this miRNA shows differential expression between healthy and failing myocardium, suggesting a functional role in the heart and more specifically in cardiac disease but the targets and functions of this novel miRNA remain to be investigated. While beyond the scope of this study, further scrutiny using validation assays in Dicer-deficient mutants could verify Dicer-dependent processing of candidate novel miRNAs and complete the definite set of miRNAs expressed -but not limited to- in the human heart.

To our knowledge, this is the first study where a deep sequencing approach was used to identify the complete set of microRNAs expressed (miRnome) in the human (failing) heart. The increased sensitivity of this method, compared to traditional ones, allowed to detect not only novel miRNAs but also the presence of miRNAs that were not previously involved in cardiac disease or neither detected in the heart. In addition, comparative profiling of hearts from patients with dilated cardiomyopathy and hearts from patients with familial hypertrophic cardiomyopathy revealed a pool of etiology-specific microRNAs which can be the basis for further research on the different pathways leading to different cardiovascular disease outcome.

Besides a main restraint of our study being the use of limited number of patients with large variation between patients and their characteristics, particularly within the DCM group, we believe that our findings can be used to generate new hypothesis and design new studies to further clarify the role of specific miRNAs, novel or not, in cardiac disease.

## Supporting Information

Table S1 Demographic/clinical characteristics and echocardiographic indices of the 16 patients included in the study.
(PDF)

Table S2 Primers sequences used for real-time PCR Analysis
(PDF)

Table S3 Quantification (normalized sequencing reads) of microRNAs in Non-Failing (NF), Dilated Cardiomyopathy (DCM) and Hypertrophic Cardiomyopathy (HCM) human hearts. *p* values for DCM versus NF, HCM vs NF and DCM vs HCM are given. Colored columns depict q values (False Discover Rate - adjusted p values) for the different comparisons. MicroRNAs that have been previously involved in cardiac disease are marked in red. MicroRNAs that are differentially expressed between DCM and HCM (based on normal p values <0.05) are depicted in purple.
(PDF)
