# Correction: A Deep Sequencing Approach to Uncover the miRNOME in the Human Heart

**DOI:** 10.1371/annotation/e33f9763-3385-42c7-b31e-d433dc8e499a

**Published:** 2013-06-18

**Authors:** Stefanos Leptidis, Hamid el Azzouzi, Sjoukje I. Lok, Roel de Weger, Servé Olieslagers, Natasja Kisters, Gustavo J. Silva, Stephane Heymans, Edwin Cuppen, Eugene Berezikov, Leon J. De Windt, Paula da Costa Martins

The name of the fifth author was spelled incorrectly. The correct name is: Servé Olieslagers. 

