# Supplementary material for: A Deep Sequencing Approach to Uncover the miRNOME in the Human Heart
Source: PLoS One. 2013 Feb 27;8(2):e57800. doi: 10.1371/journal.pone.0057800 (PMC3583901; doi:10.1371/journal.pone.0057800)
Supplement: Table S1 — Demographic/clinical characteristics and echocardiographic indices of the 16 patients included in the study. (PDF) [file pone.0057800.s001.pdf]

Supplemental Table S1: Demographic / clinical characteristics and echocardiographic indices of the 16 patients included in the study.

Parameter	NF	HCM	DCM
n	4	5	7
Demographic/Clinical aspects			
Age, years	56.4 ± 6.2	41.2 ± 5.8	50.6 ± 5.4
Men/women	2/2	2/3	5/2
Arterial hypertension, n	1	1	1
Diabetes mellitus, n	1	0	0
Medications			
β-blockers, n	1	2	5
ACEIs/ARBs, n	0	3	5
diuretics, n	0	3	6
statins, n	1	1	2
Echocardiographic indices			
LVEF (%)	nd	25 ± 4.3	18.7 ± 6.5
LVEDd (mm)	nd	61.4 ± 4.6	78.3 ± 9.7

NF - non failing; HCM - hypertrophic cardiomyopathy; DCM - dilated cardiomyopathy;
ACEIs - angiotensin converting enzyme inhibitors; ARBs - angiotensin receptor blockers; LFEF - left ventricular ejection fraction; LVEDd - left ventricular end-diastolic diameter
