# Supplementary material for: A Deep Sequencing Approach to Uncover the miRNOME in the Human Heart
Source: PLoS One. 2013 Feb 27;8(2):e57800. doi: 10.1371/journal.pone.0057800 (PMC3583901; doi:10.1371/journal.pone.0057800)
Supplement: Table S2 — Primers sequences used for real-time PCR Analysis (PDF) [file pone.0057800.s002.pdf]

Supplemental Table S2: Primers sequences used for real-time PCR analysis

Transcript	Primer Sequence
3615376	h: 5'-CAGCCCGGATCCCAGCCCACTT<br>m: 5’-CAGCCCGGATCCCAGGCCTGCG
5243049	h: 5’-CTCCTGTCATCCTGTCCAGTGTCCTCCCAG<br>m: 5’-CTCCTGTCATCCTGTATTCTGAGGTGAC
s2154	h: 5’- AGTTGCCTTTTTGTTCCCATGC<br>m: 5’- AGTTCCTTTTTGTTCCCATG
nppa	fw: 5’-CGAGGAAGTCACCATCAAACCAC<br>rv: 5’ TAGGGACAGACTGCAAGAGG
nppb	fw: 5’-AGCCTCGGAGTTGGAAACG<br>rv: 5’- CGACAGTTTGCCCTGCAAT
gapdh	fw: 5’-GAAGCTCATCTATGAGAAGGC<br>rv: 5’-AAGACGAAGGAGCTGCAGAAC
