# Supplementary material for: A Deep Sequencing Approach to Uncover the miRNOME in the Human Heart
Source: PLoS One. 2013 Feb 27;8(2):e57800. doi: 10.1371/journal.pone.0057800 (PMC3583901; doi:10.1371/journal.pone.0057800)
Supplement: Table S3 — Quantification (normalized sequencing reads) of microRNAs in Non-Failing (NF), Dilated Cardiomyopathy (DCM) and Hypertrophic Cardiomyopathy (HCM) human hearts. p values for DCM versus NF, HCM vs NF and DCM vs HCM are given. Colored columns depict q values (False Discover Rate - adjusted p values) for the different comparisons. MicroRNAs that have been previously involved in cardiac disease are marked in red. MicroRNAs that are differentially expressed between DCM and HCM (based on normal p values <0.05) are depicted in purple. (PDF) [file pone.0057800.s003.pdf]

Supplemental Table S3. Quantification (normalized sequencing reads) of microRNAs in Non-Failing (NF), Dilated Cardiomyopathy (DCM) and Hypertrophic Cardiomyopathy (HCM) human hearts. *p* values for DCM versus NF, HCM vs NF and DCM vs HCM are given. Colored columns depict q values (False Discover Rate - adjusted p values) for the different comparisons. MicroRNAs that have been previously involved in cardiac disease are marked in red. MicroRNAs that are differentially expressed between DCM andHCM (based on normal p values < 0.05) are depicted in purple.

miRNA	NF	DCM	HCM	DCM vs NF	DCM vs NF	HCM vs NF	HCM vs NF	DCM vs HCM	DCM vs HCM
hsa-mir-145 5p	19872 ± 11395	139631 ± 45998	231137 ± 20347	0.043	0.313	0.003	0.018	0.090	0.251
hsa-mir-24 3p	7332 ± 2526	78297 ± 28229	103471 ± 14515	0.051	0.313	0.017	0.041	0.430	0.609
hsa-mir-125a 5p	4853 ± 2014	28845 ± 9263	46740 ± 10224	0.085	0.313	0.009	0.030	0.161	0.342
hsa-mir-30d 5p	3984 ± 2814	16627 ± 6172	17867 ± 4892	0.135	0.313	0.126	0.153	0.870	0.931
hsa-mir-1 3p	3355 ± 1035	27862 ± 10639	70378 ± 13225	0.137	0.313	0.001	0.013	0.011	0.095
hsa-mir-23b 3p	3303 ± 1814	42036 ± 14006	31008 ± 7351	0.035	0.313	0.140	0.167	0.487	0.655
hsa-mir-1974 3p	1907 ± 1587	941 ± 266	1181 ± 371	0.361	0.460	0.518	0.532	0.805	0.878
hsa-mir-99a 5p	1838 ± 1299	6786 ± 2138	12557 ± 1854	0.106	0.313	0.004	0.021	0.049	0.184
hsa-mir-30c 5p	1834 ± 1506	8552 ± 3319	10290 ± 679	0.100	0.313	0.058	0.087	0.633	0.761
hsa-mir-133a 3p	1588 ± 1133	6950 ± 2514	21567 ± 9223	0.492	0.552	0.028	0.053	0.059	0.206
hsa-mir-1975 3p	1566 ± 1373	3638 ± 1181	5109 ± 532	0.214	0.348	0.057	0.087	0.339	0.532
hsa-mir-1978 5p	1563 ± 1178	1115 ± 237	1717 ± 179	0.563	0.612	0.852	0.858	0.409	0.599
hsa-mir-103 3p	1519 ± 700	8741 ± 3043	5829 ± 1702	0.067	0.313	0.287	0.310	0.405	0.596
hsa-mir-26a 5p	1478 ± 914	5371 ± 1817	12765 ± 1130	0.107	0.313	0.000	0.007	0.004	0.055
hsa-mir-126 3p	1432 ± 1010	6448 ± 2760	13440 ± 2177	0.177	0.328	0.007	0.026	0.053	0.190
hsa-mir-30a 5p	1255 ± 803	4300 ± 1678	7918 ± 1859	0.223	0.351	0.021	0.045	0.127	0.297
hsa-mir-1308 5p	1021 ± 837	564 ± 189	577 ± 121	0.416	0.493	0.460	0.477	0.980	0.997
hsa-mir-30b 5p	1001 ± 743	10495 ± 5091	8713 ± 488	0.116	0.313	0.224	0.249	0.741	0.832
hsa-mir-378 5p	895 ± 461	2417 ± 733	4601 ± 543	0.132	0.313	0.003	0.019	0.028	0.141
hsa-let-7f 5p	874 ± 517	3896 ± 1174	4913 ± 321	0.043	0.313	0.015	0.038	0.435	0.611
hsa-mir-29c 3p	756 ± 210	4283 ± 1784	9127 ± 3350	0.294	0.403	0.030	0.054	0.132	0.302
hsa-mir-133b 3p	755 ± 509	3694 ± 1316	11782 ± 5223	0.501	0.558	0.030	0.054	0.062	0.207

hsa-mir-451 5p	753 ± 411	3183 ± 2231	10585 ± 3011	0.484	0.548	0.017	0.041	0.035	0.153
hsa-mir-23a 3p	733 ± 264	18508 ± 6561	16694 ± 2264	0.032	0.313	0.066	0.094	0.798	0.873
hsa-let-7a 5p	727 ± 401	7693 ± 2221	11223 ± 1883	0.029	0.313	0.004	0.023	0.207	0.409
hsa-mir-378 3p	663 ± 373	1940 ± 737	3031 ± 423	0.180	0.328	0.029	0.053	0.218	0.416
hsa-mir-191 5p	654 ± 310	5515 ± 1635	9638 ± 1523	0.041	0.313	0.002	0.015	0.061	0.206
hsa-let-7g 5p	645 ± 369	3658 ± 1132	4079 ± 476	0.039	0.313	0.030	0.054	0.739	0.832
hsa-mir-29b 3p	635 ± 387	2909 ± 1257	5595 ± 1552	0.238	0.367	0.025	0.050	0.141	0.312
hsa-mir-140 3p	605 ± 238	2986 ± 911	5351 ± 759	0.062	0.313	0.002	0.016	0.049	0.184
hsa-mir-125b 5p	584 ± 308	6125 ± 1603	12202 ± 3497	0.107	0.313	0.005	0.024	0.063	0.207
hsa-mir-342 3p	523 ± 301	2851 ± 915	5094 ± 1374	0.136	0.313	0.012	0.033	0.125	0.297
hsa-let-7b 5p	505 ± 282	5066 ± 1251	6057 ± 1813	0.036	0.313	0.020	0.044	0.597	0.742
hsa-mir-107 3p	431 ± 249	2342 ± 845	1673 ± 318	0.071	0.313	0.255	0.279	0.475	0.643
hsa-mir-29a 3p	423 ± 209	2967 ± 1167	7156 ± 2547	0.295	0.403	0.018	0.042	0.076	0.231
hsa-mir-143 3p	355 ± 125	10685 ± 4338	15587 ± 2036	0.060	0.313	0.014	0.036	0.316	0.505
hsa-mir-30e 5p	332 ± 181	1705 ± 715	2486 ± 808	0.195	0.334	0.066	0.094	0.421	0.602
hsa-mir-92a 3p	297 ± 174	1715 ± 502	2765 ± 622	0.076	0.313	0.008	0.028	0.150	0.323
hsa-mir-1979 5p	280 ± 262	293 ± 69	398 ± 46	0.942	0.945	0.542	0.555	0.535	0.700
hsa-mir-223 3p	269 ± 133	3442 ± 1429	10491 ± 5385	0.483	0.548	0.048	0.076	0.109	0.276
hsa-mir-365 3p	269 ± 120	1562 ± 405	4081 ± 1269	0.247	0.373	0.005	0.024	0.025	0.141
hsa-mir-17 5p	266 ± 172	1086 ± 444	1737 ± 420	0.190	0.334	0.037	0.064	0.262	0.463
hsa-mir-193b 3p	228 ± 160	2582 ± 782	5397 ± 1556	0.133	0.313	0.006	0.025	0.061	0.206
hsa-mir-1975 5p	223 ± 190	322 ± 143	168 ± 14	0.620	0.645	0.795	0.803	0.412	0.600
hsa-mir-199a 5p	204 ± 116	3536 ± 1668	5245 ± 879	0.112	0.313	0.031	0.056	0.368	0.557
hsa-mir-100 5p	192 ± 153	561 ± 63	1486 ± 298	0.169	0.328	0.000	0.007	0.002	0.035
hsa-mir-197 3p	186 ± 74	1018 ± 242	2576 ± 488	0.093	0.313	0.000	0.007	0.003	0.049
hsa-mir-19b 3p	164 ± 95	757 ± 279	3820 ± 921	0.453	0.524	0.001	0.009	0.001	0.024
hsa-mir-1979 3p	161 ± 150	222 ± 61	274 ± 39	0.606	0.638	0.378	0.400	0.639	0.765
hsa-mir-423 3p	159 ± 102	662 ± 261	1355 ± 315	0.208	0.342	0.011	0.033	0.072	0.224
hsa-mir-26b 5p	154 ± 84	940 ± 328	2422 ± 413	0.126	0.313	0.001	0.009	0.006	0.066
hsa-mir-21 5p	153 ± 59	1454 ± 677	4779 ± 1654	0.391	0.479	0.011	0.033	0.030	0.141

hsa-mir-27b 3p	153 ± 58	1377 ± 457	1876 ± 403	0.059	0.313	0.017	0.041	0.383	0.570
hsa-let-7d 5p	150 ± 83	487 ± 120	806 ± 285	0.215	0.348	0.034	0.060	0.211	0.409
hsa-mir-152 3p	147 ± 101	443 ± 138	937 ± 102	0.125	0.313	0.001	0.013	0.012	0.095
hsa-let-7c 5p	144 ± 89	1247 ± 363	1503 ± 349	0.040	0.313	0.021	0.045	0.580	0.730
hsa-let-7e 5p	143 ± 82	1495 ± 481	1482 ± 325	0.038	0.313	0.053	0.082	0.982	0.997
hsa-mir-139 5p	137 ± 72	771 ± 468	1758 ± 415	0.316	0.423	0.027	0.051	0.105	0.270
hsa-mir-27a 3p	132 ± 51	2348 ± 823	3925 ± 1100	0.096	0.313	0.013	0.035	0.195	0.396
hsa-mir-214 3p	123 ± 63	1192 ± 302	1468 ± 261	0.016	0.313	0.006	0.026	0.462	0.636
hsa-mir-328 3p	120 ± 50	362 ± 100	752 ± 278	0.333	0.435	0.029	0.053	0.106	0.270
hsa-mir-361 5p	112 ± 57	549 ± 139	2254 ± 41	0.017	0.313	0.000	0.000	0.000	0.000
hsa-let-7i 5p	111 ± 68	898 ± 339	619 ± 117	0.062	0.313	0.241	0.264	0.454	0.629
hsa-mir-199b 5p	104 ± 60	1170 ± 557	1687 ± 301	0.128	0.313	0.042	0.070	0.415	0.600
hsa-mir-218 5p	100 ± 50	397 ± 143	613 ± 173	0.175	0.328	0.037	0.064	0.285	0.483
hsa-mir-193a 5p	97 ± 47	221 ± 70	838 ± 141	0.376	0.468	0.000	0.006	0.000	0.016
hsa-mir-361 3p	93 ± 75	207 ± 63	273 ± 29	0.208	0.342	0.071	0.097	0.420	0.602
hsa-mir-30e 3p	84 ± 61	296 ± 96	903 ± 278	0.394	0.479	0.007	0.026	0.018	0.116
hsa-mir-629 5p	81 ± 44	47 ± 34	29 ± 8	0.480	0.547	0.319	0.341	0.690	0.807
hsa-mir-660 5p	79 ± 59	137 ± 61	254 ± 106	0.610	0.639	0.164	0.192	0.280	0.479
hsa-mir-29b 5p	77 ± 49	232 ± 83	303 ± 43	0.149	0.319	0.057	0.087	0.466	0.637
hsa-mir-185 5p	74 ± 42	328 ± 114	266 ± 54	0.082	0.313	0.207	0.235	0.630	0.761
hsa-mir-532 5p	71 ± 45	196 ± 66	241 ± 45	0.159	0.328	0.081	0.106	0.580	0.730
hsa-mir-340 3p	68 ± 45	177 ± 71	343 ± 58	0.267	0.381	0.017	0.041	0.080	0.236
hsa-mir-222 3p	68 ± 37	295 ± 75	634 ± 172	0.171	0.328	0.005	0.024	0.037	0.159
hsa-mir-193b 5p	67 ± 43	96 ± 21	164 ± 16	0.430	0.503	0.026	0.050	0.066	0.210
hsa-mir-30a 3p	63 ± 45	372 ± 146	872 ± 319	0.310	0.418	0.022	0.046	0.090	0.251
hsa-mir-1974 5p	62 ± 48	151 ± 39	122 ± 31	0.141	0.314	0.338	0.359	0.601	0.743
hsa-mir-887 3p	58 ± 43	181 ± 60	232 ± 21	0.115	0.313	0.044	0.071	0.467	0.637
hsa-mir-455 3p	56 ± 24	1352 ± 485	1770 ± 356	0.050	0.313	0.019	0.043	0.470	0.639
hsa-mir-22 3p	56 ± 36	1331 ± 522	1226 ± 162	0.049	0.313	0.086	0.112	0.851	0.916
hsa-mir-664 3p	56 ± 22	433 ± 126	929 ± 98	0.033	0.313	0.000	0.006	0.005	0.066

hsa-mir-199a 3p	56 ± 17	1484 ± 697	2838 ± 515	0.122	0.313	0.010	0.031	0.117	0.287
hsa-mir-130a 3p	55 ± 42	229 ± 84	333 ± 80	0.149	0.319	0.040	0.067	0.350	0.544
hsa-mir-29c 5p	55 ± 36	159 ± 63	564 ± 135	0.420	0.496	0.002	0.016	0.004	0.059
hsa-mir-574 3p	53 ± 19	1035 ± 312	1588 ± 441	0.062	0.313	0.010	0.032	0.240	0.438
hsa-mir-148a 3p	53 ± 31	192 ± 105	371 ± 27	0.262	0.381	0.026	0.051	0.131	0.302
hsa-mir-146b 5p	52 ± 42	120 ± 42	443 ± 250	0.736	0.751	0.087	0.112	0.103	0.270
hsa-mir-181a 5p	47 ± 38	1006 ± 367	892 ± 145	0.040	0.313	0.083	0.108	0.776	0.865
hsa-mir-505 3p	46 ± 31	158 ± 50	266 ± 103	0.274	0.384	0.056	0.087	0.260	0.463
hsa-mir-574 5p	44 ± 32	136 ± 39	225 ± 38	0.118	0.313	0.009	0.030	0.111	0.276
hsa-mir-1826 5p	42 ± 23	267 ± 130	228 ± 92	0.183	0.328	0.299	0.321	0.795	0.873
hsa-mir-99b 3p	41 ± 32	102 ± 48	183 ± 46	0.373	0.467	0.064	0.094	0.209	0.409
hsa-mir-301a 3p	39 ± 29	79 ± 23	168 ± 35	0.349	0.446	0.011	0.032	0.034	0.152
hsa-mir-27b 5p	38 ± 33	147 ± 47	171 ± 34	0.099	0.313	0.065	0.094	0.687	0.807
hsa-mir-886 5p	38 ± 34	52 ± 19	56 ± 20	0.684	0.705	0.628	0.639	0.904	0.955
hsa-mir-590 5p	37 ± 26	181 ± 82	306 ± 84	0.222	0.351	0.043	0.070	0.253	0.456
hsa-mir-421 3p	36 ± 27	99 ± 31	128 ± 29	0.175	0.328	0.073	0.099	0.498	0.662
hsa-mir-210 3p	34 ± 24	138 ± 43	378 ± 97	0.264	0.381	0.003	0.019	0.013	0.096
hsa-mir-192 5p	34 ± 16	144 ± 54	254 ± 48	0.140	0.314	0.012	0.033	0.119	0.287
hsa-mir-182 5p	33 ± 16	24 ± 10	33 ± 9	0.599	0.633	0.992	0.992	0.567	0.721
hsa-mir-125b 3p	31 ± 20	241 ± 81	335 ± 33	0.043	0.313	0.009	0.030	0.298	0.491
hsa-mir-10b 5p	30 ± 19	116 ± 40	161 ± 33	0.123	0.313	0.035	0.062	0.375	0.565
hsa-mir-10a 5p	29 ± 18	52 ± 18	83 ± 16	0.394	0.479	0.077	0.102	0.231	0.429
hsa-mir-181b 5p	29 ± 18	281 ± 103	115 ± 40	0.052	0.313	0.511	0.526	0.156	0.333
hsa-mir-425 5p	29 ± 19	142 ± 59	269 ± 116	0.327	0.432	0.063	0.092	0.239	0.438
hsa-mir-199b 3p	28 ± 8	742 ± 349	1417 ± 258	0.122	0.313	0.010	0.031	0.118	0.287
hsa-mir-15b 5p	26 ± 8	552 ± 202	855 ± 164	0.059	0.313	0.009	0.030	0.225	0.420
hsa-mir-25 3p	26 ± 18	174 ± 74	346 ± 51	0.128	0.313	0.006	0.025	0.064	0.207
hsa-mir-374b 5p	25 ± 14	112 ± 67	381 ± 148	0.527	0.579	0.028	0.053	0.053	0.190
hsa-mir-628 5p	23 ± 13	50 ± 15	68 ± 12	0.225	0.353	0.068	0.095	0.377	0.565
hsa-mir-132 3p	23 ± 12	141 ± 66	430 ± 119	0.330	0.435	0.006	0.026	0.020	0.119

hsa-mir-320b 3p	23 ± 20	280 ± 87	220 ± 47	0.026	0.313	0.096	0.121	0.541	0.700
hsa-mir-574 3p	22 ± 18	57 ± 13	105 ± 18	0.153	0.321	0.005	0.024	0.042	0.171
hsa-mir-331 3p	22 ± 8	1998 ± 644	4086 ± 743	0.048	0.313	0.001	0.013	0.028	0.141
hsa-mir-133a 5p	22 ± 10	91 ± 33	227 ± 47	0.199	0.336	0.002	0.016	0.013	0.096
hsa-mir-320a 3p	22 ± 11	211 ± 61	244 ± 82	0.060	0.313	0.042	0.070	0.715	0.825
hsa-mir-34a 5p	21 ± 19	407 ± 172	409 ± 167	0.116	0.313	0.138	0.166	0.993	0.997
hsa-mir-486-as 5p	21 ± 17	174 ± 54	349 ± 42	0.042	0.313	0.001	0.008	0.016	0.110
hsa-mir-486 5p	21 ± 17	174 ± 54	349 ± 42	0.042	0.313	0.001	0.008	0.016	0.110
hsa-mir-210 5p	20 ± 9	81 ± 25	129 ± 22	0.089	0.313	0.009	0.029	0.142	0.312
hsa-mir-874 5p	20 ± 12	115 ± 43	241 ± 48	0.137	0.313	0.004	0.023	0.043	0.173
hsa-mir-28 3p	20 ± 8	75 ± 25	129 ± 21	0.121	0.313	0.009	0.029	0.103	0.270
hsa-mir-195 5p	20 ± 13	156 ± 50	235 ± 30	0.043	0.313	0.005	0.025	0.186	0.381
hsa-mir-409 3p	18 ± 10	207 ± 86	292 ± 134	0.199	0.336	0.089	0.113	0.523	0.692
hsa-mir-320c 3p	18 ± 17	79 ± 21	109 ± 21	0.067	0.313	0.014	0.036	0.294	0.491
hsa-mir-92b 3p	18 ± 8	138 ± 24	478 ± 109	0.193	0.334	0.000	0.007	0.001	0.024
hsa-mir-224 5p	18 ± 11	317 ± 99	424 ± 131	0.064	0.313	0.023	0.047	0.453	0.629
hsa-mir-345 5p	17 ± 14	105 ± 32	191 ± 36	0.075	0.313	0.003	0.020	0.064	0.207
hsa-mir-151 5p	17 ± 7	185 ± 69	186 ± 53	0.073	0.313	0.089	0.113	0.986	0.997
hsa-mir-574 5p	17 ± 12	32 ± 11	40 ± 7	0.340	0.442	0.184	0.214	0.595	0.742
hsa-mir-494 3p	16 ± 9	135 ± 51	213 ± 62	0.131	0.313	0.027	0.052	0.281	0.479
hsa-mir-151 3p	16 ± 8	148 ± 50	156 ± 40	0.056	0.313	0.058	0.088	0.895	0.952
hsa-mir-629 3p	16 ± 6	10 ± 7	7 ± 3	0.585	0.624	0.388	0.409	0.679	0.801
hsa-mir-1307 3p	15 ± 14	54 ± 21	106 ± 46	0.381	0.469	0.068	0.095	0.215	0.414
hsa-mir-19a 3p	15 ± 11	33 ± 9	162 ± 36	0.555	0.605	0.000	0.008	0.000	0.023
hsa-mir-151 5p	15 ± 9	70 ± 24	109 ± 24	0.112	0.313	0.018	0.042	0.222	0.419
hsa-mir-486 3p	14 ± 4	173 ± 66	148 ± 28	0.055	0.313	0.122	0.149	0.728	0.829
hsa-mir-1287 5p	14 ± 10	43 ± 13	48 ± 4	0.103	0.313	0.072	0.099	0.724	0.828
hsa-mir-499 5p	14 ± 10	190 ± 99	1086 ± 386	0.585	0.624	0.007	0.026	0.009	0.082
hsa-mir-501 5p	14 ± 11	49 ± 16	86 ± 22	0.185	0.328	0.019	0.043	0.141	0.312
hsa-mir-362 5p	13 ± 11	31 ± 11	83 ± 24	0.453	0.524	0.014	0.036	0.030	0.141

hsa-mir-1978 3p	12 ± 5	56 ± 18	123 ± 28	0.161	0.328	0.004	0.021	0.030	0.141
hsa-mir-423 5p	12 ± 7	67 ± 18	173 ± 22	0.063	0.313	0.000	0.005	0.001	0.024
hsa-mir-1226 3p	12 ± 8	27 ± 5	68 ± 10	0.195	0.334	0.000	0.007	0.001	0.026
hsa-mir-744 5p	12 ± 4	118 ± 51	330 ± 62	0.172	0.328	0.001	0.013	0.008	0.080
hsa-mir-146a 5p	11 ± 7	61 ± 20	115 ± 19	0.085	0.313	0.003	0.019	0.048	0.184
hsa-let-7b 3p	11 ± 3	77 ± 16	200 ± 22	0.019	0.313	0.000	0.001	0.000	0.009
hsa-mir-483 3p	11 ± 4	146 ± 48	613 ± 191	0.400	0.484	0.003	0.019	0.006	0.066
hsa-mir-1306 5p	11 ± 4	73 ± 16	134 ± 54	0.184	0.328	0.023	0.046	0.171	0.357
hsa-mir-302d 3p	11 ± 7	25 ± 12	33 ± 6	0.343	0.444	0.178	0.208	0.575	0.729
hsa-mir-95 3p	11 ± 7	78 ± 27	97 ± 15	0.052	0.313	0.024	0.049	0.539	0.700
hsa-mir-128 3p	10 ± 4	156 ± 42	372 ± 37	0.018	0.313	0.000	0.002	0.001	0.024
hsa-mir-221 3p	10 ± 3	102 ± 56	122 ± 59	0.251	0.373	0.197	0.227	0.792	0.872
hsa-mir-320d 3p	10 ± 8	159 ± 54	211 ± 48	0.051	0.313	0.018	0.042	0.443	0.618
hsa-mir-7 3p	10 ± 3	145 ± 53	283 ± 74	0.121	0.313	0.008	0.028	0.092	0.251
hsa-mir-16 5p	10 ± 6	54 ± 25	63 ± 4	0.139	0.314	0.100	0.125	0.737	0.832
hsa-mir-486-as 3p	9 ± 4	104 ± 39	98 ± 17	0.054	0.313	0.088	0.113	0.880	0.938
hsa-mir-135a 5p	9 ± 4	25 ± 9	65 ± 11	0.243	0.369	0.001	0.014	0.006	0.066
hsa-mir-20a 5p	9 ± 7	31 ± 11	113 ± 26	0.379	0.469	0.001	0.013	0.003	0.049
hsa-mir-98 5p	8 ± 6	88 ± 29	87 ± 13	0.035	0.313	0.048	0.076	0.980	0.997
hsa-mir-93 3p	8 ± 6	69 ± 26	118 ± 17	0.076	0.313	0.007	0.026	0.127	0.297
hsa-mir-181a 3p	8 ± 6	119 ± 31	294 ± 73	0.112	0.313	0.001	0.013	0.012	0.096
hsa-let-7f 3p	8 ± 3	29 ± 5	90 ± 12	0.085	0.313	0.000	0.001	0.000	0.006
hsa-mir-99a 3p	8 ± 5	15 ± 9	38 ± 12	0.612	0.639	0.066	0.094	0.106	0.270
hsa-mir-338-as 5p	8 ± 7	6 ± 2	14 ± 6	0.861	0.869	0.429	0.447	0.280	0.479
hsa-mir-208a 5p	7 ± 4	141 ± 57	292 ± 61	0.110	0.313	0.005	0.024	0.059	0.206
hsa-mir-140 5p	7 ± 3	92 ± 36	129 ± 31	0.094	0.313	0.029	0.054	0.405	0.596
hsa-mir-425 3p	7 ± 4	69 ± 23	97 ± 26	0.077	0.313	0.021	0.045	0.364	0.556
hsa-mir-766 3p	7 ± 3	36 ± 6	82 ± 17	0.073	0.313	0.000	0.007	0.005	0.066
hsa-mir-342 5p	7 ± 6	28 ± 8	40 ± 3	0.066	0.313	0.013	0.035	0.276	0.479
hsa-let-7a 3p	7 ± 3	38 ± 13	121 ± 20	0.149	0.319	0.000	0.006	0.001	0.024

hsa-mir-664 5p	7 ± 4	13 ± 3	28 ± 3	0.285	0.394	0.005	0.024	0.020	0.119
hsa-mir-186 5p	7 ± 5	166 ± 66	167 ± 21	0.052	0.313	0.065	0.094	0.987	0.997
hsa-mir-15a 5p	6 ± 4	48 ± 20	78 ± 11	0.103	0.313	0.014	0.036	0.198	0.400
hsa-mir-452 5p	6 ± 5	59 ± 21	58 ± 7	0.045	0.313	0.060	0.089	0.989	0.997
hsa-mir-338 5p	6 ± 4	18 ± 6	55 ± 21	0.497	0.556	0.021	0.045	0.041	0.169
hsa-mir-663b 3p	6 ± 3	13 ± 5	17 ± 3	0.321	0.427	0.153	0.179	0.537	0.700
hsa-mir-652 5p	6 ± 4	20 ± 8	20 ± 5	0.195	0.334	0.224	0.249	1.000	1.000
hsa-mir-497 5p	6 ± 1	52 ± 16	142 ± 41	0.222	0.351	0.004	0.021	0.020	0.119
hsa-mir-20b 5p	6 ± 3	27 ± 10	20 ± 6	0.124	0.313	0.312	0.335	0.588	0.737
hsa-mir-144 5p	6 ± 3	8 ± 5	22 ± 5	0.810	0.823	0.079	0.105	0.077	0.231
hsa-mir-382 5p	6 ± 4	53 ± 18	83 ± 27	0.129	0.313	0.027	0.052	0.290	0.488
hsa-mir-221 5p	6 ± 2	32 ± 10	39 ± 5	0.047	0.313	0.022	0.046	0.544	0.702
hsa-mir-187 3p	6 ± 4	10 ± 5	11 ± 8	0.590	0.625	0.563	0.574	0.931	0.978
hsa-mir-127 3p	5 ± 2	107 ± 66	114 ± 43	0.227	0.355	0.230	0.253	0.933	0.978
hsa-mir-1259 5p	5 ± 5	10 ± 6	4 ± 2	0.575	0.622	0.872	0.875	0.436	0.611
hsa-let-7d 3p	5 ± 4	64 ± 24	244 ± 84	0.410	0.489	0.007	0.026	0.016	0.110
hsa-mir-93 5p	5 ± 3	15 ± 5	31 ± 8	0.270	0.382	0.018	0.042	0.085	0.244
hsa-mir-505 5p	5 ± 4	12 ± 3	18 ± 4	0.262	0.381	0.066	0.094	0.317	0.505
hsa-mir-106b 5p	5 ± 2	58 ± 20	275 ± 76	0.403	0.484	0.001	0.013	0.002	0.045
hsa-mir-148b 3p	5 ± 2	42 ± 16	76 ± 14	0.097	0.313	0.007	0.027	0.111	0.276
hsa-mir-1249 3p	5 ± 2	34 ± 8	72 ± 18	0.094	0.313	0.002	0.016	0.027	0.141
hsa-mir-130b 3p	4 ± 4	14 ± 5	19 ± 6	0.251	0.373	0.117	0.144	0.537	0.700
hsa-mir-339 5p	4 ± 3	39 ± 16	105 ± 31	0.254	0.375	0.006	0.026	0.030	0.141
hsa-mir-155 5p	4 ± 3	10 ± 3	24 ± 5	0.364	0.460	0.011	0.032	0.033	0.148
hsa-mir-642 5p	4 ± 2	7 ± 2	37 ± 5	0.587	0.624	0.000	0.005	0.000	0.006
hsa-mir-379 5p	4 ± 3	39 ± 18	43 ± 5	0.121	0.313	0.104	0.130	0.825	0.893
hsa-mir-181d 5p	4 ± 2	36 ± 12	21 ± 7	0.041	0.313	0.268	0.291	0.295	0.491
hsa-mir-532 3p	4 ± 3	19 ± 7	48 ± 17	0.331	0.435	0.018	0.042	0.067	0.211
hsa-mir-502 3p	4 ± 2	15 ± 6	25 ± 9	0.277	0.387	0.061	0.090	0.278	0.479
hsa-mir-28 5p	4 ± 2	140 ± 55	186 ± 41	0.064	0.313	0.026	0.050	0.489	0.655

hsa-mir-130b 5p	4 ± 1	43 ± 17	105 ± 50	0.372	0.467	0.043	0.071	0.140	0.312
hsa-mir-296 5p	4 ± 2	75 ± 22	197 ± 49	0.132	0.313	0.001	0.013	0.011	0.095
hsa-mir-1271 5p	4 ± 2	57 ± 22	79 ± 15	0.067	0.313	0.022	0.046	0.416	0.600
hsa-mir-1301 3p	4 ± 3	36 ± 13	53 ± 10	0.066	0.313	0.012	0.034	0.262	0.463
hsa-mir-590 3p	4 ± 2	21 ± 10	38 ± 7	0.176	0.328	0.022	0.046	0.176	0.364
hsa-mir-1180 3p	4 ± 2	19 ± 6	45 ± 12	0.204	0.340	0.005	0.024	0.032	0.145
hsa-mir-30b 3p	4 ± 2	11 ± 4	33 ± 5	0.273	0.384	0.001	0.013	0.005	0.064
hsa-mir-487b 3p	3 ± 2	82 ± 31	175 ± 55	0.172	0.328	0.011	0.033	0.090	0.251
hsa-mir-145 3p	3 ± 2	55 ± 23	83 ± 6	0.062	0.313	0.012	0.033	0.263	0.463
hsa-mir-125a 3p	3 ± 1	29 ± 12	44 ± 3	0.079	0.313	0.014	0.036	0.245	0.444
hsa-mir-138 5p	3 ± 3	25 ± 11	21 ± 4	0.101	0.313	0.210	0.236	0.713	0.825
hsa-mir-103 5p	3 ± 3	21 ± 8	28 ± 9	0.169	0.328	0.080	0.105	0.549	0.705
hsa-mir-500 3p	3 ± 2	12 ± 4	21 ± 6	0.264	0.381	0.044	0.072	0.219	0.416
hsa-mir-7 5p	3 ± 2	6 ± 2	12 ± 6	0.638	0.662	0.203	0.231	0.327	0.516
hsa-mir-625-as 3p	3 ± 1	37 ± 11	72 ± 21	0.127	0.313	0.008	0.028	0.093	0.251
hsa-mir-625 3p	3 ± 1	37 ± 11	72 ± 21	0.127	0.313	0.008	0.028	0.093	0.251
hsa-mir-101 3p	3 ± 1	129 ± 78	213 ± 41	0.192	0.334	0.052	0.081	0.347	0.542
hsa-mir-302b 3p	3 ± 3	14 ± 6	14 ± 3	0.174	0.328	0.209	0.236	0.980	0.997
hsa-mir-224 3p	3 ± 1	46 ± 16	77 ± 13	0.055	0.313	0.005	0.024	0.132	0.302
hsa-mir-495 3p	3 ± 1	35 ± 12	76 ± 30	0.242	0.369	0.022	0.046	0.120	0.288
hsa-mir-940 3p	3 ± 2	25 ± 7	61 ± 13	0.103	0.313	0.001	0.011	0.009	0.083
hsa-mir-598 3p	3 ± 1	23 ± 12	27 ± 9	0.182	0.328	0.143	0.170	0.788	0.871
hsa-mir-493 5p	3 ± 1	7 ± 2	23 ± 6	0.539	0.589	0.008	0.028	0.013	0.096
hsa-mir-181c 3p	3 ± 1	24 ± 13	17 ± 2	0.169	0.328	0.380	0.401	0.616	0.755
hsa-mir-491 5p	3 ± 2	18 ± 7	14 ± 2	0.107	0.313	0.225	0.249	0.704	0.820
hsa-mir-1247 5p	3 ± 2	16 ± 5	87 ± 44	0.690	0.709	0.037	0.064	0.045	0.175
hsa-mir-584 5p	3 ± 1	14 ± 5	52 ± 9	0.235	0.364	0.000	0.006	0.001	0.024
hsa-mir-338 3p	3 ± 2	12 ± 6	53 ± 10	0.348	0.446	0.000	0.007	0.001	0.024
hsa-mir-362 3p	3 ± 2	10 ± 4	34 ± 11	0.452	0.524	0.012	0.034	0.027	0.141
hsa-mir-500 5p	3 ± 2	8 ± 2	22 ± 7	0.402	0.484	0.016	0.041	0.044	0.174

hsa-mir-550 3p	3 ± 2	37 ± 17	47 ± 13	0.137	0.313	0.076	0.102	0.615	0.755
hsa-mir-616 5p	2 ± 2	16 ± 7	19 ± 5	0.185	0.328	0.137	0.165	0.756	0.845
hsa-mir-26b 3p	2 ± 2	28 ± 15	44 ± 8	0.160	0.328	0.045	0.073	0.367	0.557
hsa-mir-181c 5p	2 ± 1	24 ± 9	29 ± 5	0.074	0.313	0.046	0.074	0.666	0.788
hsa-mir-30c 3p	2 ± 1	20 ± 7	23 ± 5	0.081	0.313	0.058	0.087	0.731	0.829
hsa-mir-323 3p	2 ± 1	17 ± 7	21 ± 7	0.154	0.321	0.092	0.116	0.652	0.777
hsa-mir-106a 5p	2 ± 1	15 ± 4	27 ± 9	0.171	0.328	0.025	0.049	0.200	0.400
hsa-mir-139 3p	2 ± 2	5 ± 3	18 ± 8	0.699	0.715	0.077	0.102	0.099	0.266
hsa-mir-760 3p	2 ± 1	4 ± 2	17 ± 3	0.648	0.671	0.007	0.027	0.008	0.077
hsa-mir-326 3p	2 ± 1	42 ± 22	152 ± 43	0.349	0.446	0.005	0.024	0.013	0.096
hsa-mir-330 3p	2 ± 1	14 ± 5	37 ± 14	0.364	0.460	0.021	0.045	0.068	0.212
hsa-mir-550 5p	2 ± 1	12 ± 4	12 ± 5	0.152	0.321	0.199	0.229	0.937	0.979
hsa-mir-708 5p	2 ± 1	26 ± 9	24 ± 7	0.065	0.313	0.110	0.137	0.853	0.916
hsa-mir-16 3p	2 ± 2	23 ± 12	24 ± 8	0.177	0.328	0.186	0.215	0.943	0.979
hsa-mir-17 3p	2 ± 1	23 ± 9	21 ± 6	0.094	0.313	0.139	0.167	0.905	0.955
hsa-mir-374a 5p	2 ± 2	11 ± 6	33 ± 11	0.416	0.493	0.020	0.045	0.054	0.192
hsa-mir-432 5p	2 ± 1	5 ± 2	10 ± 3	0.523	0.576	0.142	0.169	0.297	0.491
hsa-mir-31 5p	2 ± 1	13 ± 6	89 ± 68	0.829	0.840	0.143	0.170	0.146	0.315
hsa-mir-29a 5p	2 ± 1	10 ± 5	9 ± 3	0.241	0.369	0.298	0.321	0.942	0.979
hsa-mir-337 5p	2 ± 1	8 ± 5	18 ± 7	0.459	0.527	0.089	0.113	0.221	0.419
hsa-mir-483 5p	2 ± 1	7 ± 1	20 ± 4	0.269	0.382	0.002	0.015	0.006	0.066
hsa-mir-542 3p	2 ± 1	36 ± 18	63 ± 19	0.185	0.328	0.037	0.064	0.266	0.466
hsa-mir-337 3p	2 ± 1	30 ± 12	68 ± 22	0.203	0.340	0.012	0.033	0.078	0.231
hsa-mir-132 5p	2 ± 1	29 ± 16	49 ± 13	0.200	0.336	0.046	0.074	0.303	0.496
hsa-mir-671 3p	2 ± 1	23 ± 7	41 ± 6	0.049	0.313	0.002	0.016	0.064	0.207
hsa-mir-212 3p	2 ± 1	20 ± 9	74 ± 26	0.424	0.497	0.011	0.032	0.026	0.141
hsa-mir-15b 3p	2 ± 2	17 ± 7	15 ± 5	0.112	0.313	0.201	0.230	0.782	0.868
hsa-mir-129 3p	2 ± 1	13 ± 4	16 ± 3	0.048	0.313	0.029	0.054	0.654	0.777
hsa-mir-218 3p	2 ± 0	9 ± 3	32 ± 8	0.377	0.468	0.003	0.019	0.007	0.066
hsa-mir-1227 3p	2 ± 1	7 ± 2	13 ± 4	0.266	0.381	0.036	0.063	0.177	0.364

hsa-mir-652 3p	2 ± 2	1 ± 1	30 ± 17	0.996	0.996	0.068	0.095	0.040	0.166
hsa-mir-874 3p	1 ± 1	40 ± 15	24 ± 9	0.057	0.313	0.268	0.291	0.384	0.570
hsa-mir-424 5p	1 ± 1	36 ± 16	36 ± 8	0.090	0.313	0.113	0.139	0.991	0.997
hsa-mir-671 5p	1 ± 1	29 ± 10	12 ± 3	0.028	0.313	0.393	0.412	0.127	0.297
hsa-mir-675 3p	1 ± 1	23 ± 10	37 ± 11	0.148	0.319	0.035	0.062	0.317	0.505
hsa-mir-214 5p	1 ± 1	15 ± 7	20 ± 4	0.122	0.313	0.068	0.095	0.623	0.760
hsa-mir-455 5p	1 ± 1	14 ± 5	16 ± 4	0.105	0.313	0.073	0.100	0.720	0.826
hsa-mir-23b 5p	1 ± 1	12 ± 3	34 ± 12	0.300	0.405	0.009	0.030	0.035	0.153
hsa-mir-935 3p	1 ± 1	11 ± 5	33 ± 9	0.286	0.394	0.007	0.026	0.027	0.141
hsa-mir-550 3p	1 ± 1	18 ± 8	23 ± 7	0.147	0.319	0.083	0.108	0.625	0.760
hsa-mir-424 3p	1 ± 1	25 ± 15	10 ± 6	0.182	0.328	0.641	0.650	0.353	0.545
hsa-mir-744 3p	1 ± 1	22 ± 9	38 ± 6	0.079	0.313	0.008	0.028	0.145	0.315
hsa-mir-92a 5p	1 ± 1	12 ± 3	4 ± 1	0.028	0.313	0.505	0.522	0.088	0.250
hsa-mir-188 5p	1 ± 1	17 ± 8	15 ± 5	0.131	0.313	0.219	0.246	0.810	0.880
hsa-mir-504 5p	1 ± 1	16 ± 5	40 ± 9	0.144	0.319	0.002	0.016	0.020	0.119
hsa-mir-490 3p	1 ± 1	14 ± 7	9 ± 5	0.169	0.328	0.418	0.437	0.560	0.716
hsa-mir-99b 5p	1 ± 1	12 ± 5	18 ± 4	0.118	0.313	0.028	0.052	0.316	0.505
hsa-mir-376c 3p	1 ± 0	21 ± 8	25 ± 6	0.088	0.313	0.061	0.090	0.716	0.825
hsa-mir-363 3p	1 ± 0	11 ± 4	23 ± 7	0.211	0.345	0.016	0.041	0.106	0.270
hsa-let-7c 3p	1 ± 1	9 ± 3	22 ± 5	0.163	0.328	0.003	0.021	0.028	0.141
hsa-mir-130a 5p	1 ± 1	8 ± 4	14 ± 3	0.247	0.373	0.062	0.091	0.318	0.505
hsa-mir-107 5p	1 ± 1	8 ± 3	14 ± 3	0.219	0.351	0.038	0.065	0.232	0.429
hsa-mir-1228 3p	1 ± 1	8 ± 3	34 ± 10	0.462	0.528	0.004	0.021	0.007	0.066
rno-mir-320 3p	1 ± 1	7 ± 3	13 ± 2	0.251	0.373	0.040	0.067	0.210	0.409
hsa-mir-101 5p	1 ± 1	7 ± 5	12 ± 7	0.490	0.552	0.223	0.249	0.494	0.660
hsa-mir-208b 5p	1 ± 1	5 ± 3	24 ± 8	0.580	0.623	0.013	0.035	0.018	0.118
hsa-mir-106b 3p	1 ± 1	5 ± 3	12 ± 4	0.457	0.527	0.057	0.087	0.142	0.312
hsa-mir-2110 5p	1 ± 0	66 ± 26	66 ± 28	0.091	0.313	0.113	0.139	0.993	0.997
hsa-mir-329 3p	1 ± 0	30 ± 13	48 ± 23	0.221	0.351	0.077	0.102	0.425	0.604
hsa-mir-382 3p	1 ± 0	13 ± 5	33 ± 13	0.318	0.425	0.021	0.045	0.082	0.238

hsa-mir-1248 5p	1 ± 0	10 ± 4	14 ± 8	0.260	0.381	0.150	0.177	0.632	0.761
hsa-mir-769 5p	1 ± 1	8 ± 4	43 ± 12	0.511	0.568	0.002	0.016	0.003	0.050
hsa-mir-369 5p	1 ± 0	7 ± 3	12 ± 4	0.299	0.405	0.076	0.102	0.313	0.505
hsa-mir-23a 5p	1 ± 0	6 ± 3	26 ± 11	0.578	0.623	0.025	0.050	0.040	0.166
hsa-mir-190 3p	1 ± 0	6 ± 2	16 ± 3	0.285	0.394	0.006	0.025	0.022	0.130
hsa-mir-154 5p	1 ± 0	5 ± 3	19 ± 6	0.520	0.575	0.016	0.041	0.030	0.141
hsa-mir-142 5p	1 ± 0	28 ± 13	49 ± 11	0.123	0.313	0.017	0.041	0.200	0.400
hsa-let-7e 3p	1 ± 0	17 ± 5	35 ± 10	0.127	0.313	0.006	0.026	0.073	0.224
hsa-mir-24 5p	1 ± 0	12 ± 3	17 ± 3	0.029	0.313	0.008	0.028	0.354	0.545
hsa-mir-409 5p	1 ± 0	5 ± 2	12 ± 4	0.407	0.487	0.058	0.087	0.167	0.351
hsa-mir-18a 5p	1 ± 1	2 ± 1	29 ± 17	0.909	0.915	0.067	0.095	0.050	0.184
